# The International Hahnemannian Association

**Published:** 1897-05

**Authors:** 


The International Hahnemannian Association will hold its eighteenth annual meeting, beginning on Tuesday morning, June 29th, at 11 o'clock, at the International Hotel, Niagara Falls. Through the American Institute of Homeopathy a large reduction in railroad fare may be obtained by purchasing a full fare ticket to Buffalo, and securing from the ticket agent a certificate of purchase, which, on being presented to the Transportation Agent of the A. I. H., will secure a one-third rate of fare on return trip.

